# Heat Stress Causes Spatially-Distinct Membrane Re-Modelling in K562 Leukemia Cells

**DOI:** 10.1371/journal.pone.0021182

**Published:** 2011-06-16

**Authors:** Gábor Balogh, Giuseppe Maulucci, Imre Gombos, Ibolya Horváth, Zsolt Török, Mária Péter, Elfrieda Fodor, Tibor Páli, Sándor Benkő, Tiziana Parasassi, Marco De Spirito, John L. Harwood, László Vígh

**Affiliations:** 1 Institute of Biochemistry, Biological Research Centre, Hungarian Academy of Sciences, Szeged, Hungary; 2 Institute of Biophysics, Biological Research Centre, Hungarian Academy of Sciences, Szeged, Hungary; 3 First Department of Internal Medicine, Albert Szent-Györgyi Clinical Center, University of Szeged, Szeged, Hungary; 4 Institute of Translational Pharmacology, CNR, Rome, Italy; 5 Istituto di Fisica, Universitá Cattolica Sacro Cuore, Rome, Italy; 6 School of Biosciences, Cardiff University, Cardiff, Wales, United Kingdom; Semmelweis University, Hungary

## Abstract

Cellular membranes respond rapidly to various environmental perturbations. Previously we showed that modulations in membrane fluidity achieved by heat stress (HS) resulted in pronounced membrane organization alterations which could be intimately linked to the expression and cellular distribution of heat shock proteins. Here we examine heat-induced membrane changes using several visualisation methods. With Laurdan two-photon microscopy we demonstrate that, in contrast to the enhanced formation of ordered domains in surface membranes, the molecular disorder is significantly elevated within the internal membranes of cells preexposed to mild HS. These results were compared with those obtained by anisotropy, fluorescence lifetime and electron paramagnetic resonance measurements. All probes detected membrane changes upon HS. However, the structurally different probes revealed substantially distinct alterations in membrane heterogeneity. These data call attention to the careful interpretation of results obtained with only a single label. Subtle changes in membrane microstructure in the decision-making of thermal cell killing could have potential application in cancer therapy.

## Introduction

There has been a renewed interest in the application of hyperthermia in antitumor therapy. This is a promising treatment modality for cancer, especially in combination with radiotherapy, because various tumors are often more thermally sensitive than normal tissues [1]. However, the primary target of cellular heat killing is still unknown. It was suggested 30 years ago that membranes of tumor cells are the major targets for heat treatment and that the effectiveness of hyperthermic cell killing is influenced basically by the fluidity of membranes [2]. In support of this proposal, the use of membrane fluidizing agents (typically local anaesthetics) was shown to potentiate the therapeutic effect of hyperthermia [2].

On the other hand, cells exposed to non-lethal elevated temperatures or treated with various substances targeting membranes develop a stronger resistance to a subsequent severe heat stress (HS) [3] – a phenomenon called acquired thermotolerance. One of the major obstacles for many types of anticancer therapy is that they induce a stress response (also called a heat shock response) making tumors more resistant to subsequent treatments. Amongst other effects, the heat shock response restores the normal protein folding environment by upregulating heat shock proteins (Hsps) and changing their cellular locations [4]–[7] thus altering pathways controlling cell survival, growth and metabolism.

The question as to how membrane structural alterations can be linked to the Janus-like properties of hyperthermia in cancer therapy has been addressed in recent reviews [1], [8]. Cellular membranes have also been implicated as the primary heat sensors [9], [10] as well as in the decision-making for thermal cell killing [1], [11].

Previously we demonstrated that membrane hyperfluidization acts as a primary signal to initiate the Hsp response in prokaryotic organisms [12], [13], yeast [14], K562 leukemia [15] and B16 melanoma cells [16]. Similarly to HS, exposing K562 cells to benzyl alcohol (BA), which is a well-established membrane fluidizing agent [17], [18], elicited nearly identical increases in cytosolic Ca^2+^ concentration, Hsp70 synthesis and mitochondrial hyperpolarization [15]. Moreover, the microdomain organization of plasma membranes (PM) has been shown to be a decisive factor in the perception and transduction of heat- or non-proteotoxic chemical agent-induced membrane stress into signals which then trigger the transcriptional activation of heat shock genes in B16 melanoma cells [16]. As reviewed recently, the temporal and spatial regulation of the membrane hyperfine structure appears to be a hallmark of cellular stress sensing and signalling events [6], [9].

HS causes a change in the physical state of membranes in the post-heat phase [19]–[21]. Moreover, it is well established that phospholipases and sphingomyelinases are activated during various stresses thus cleaving the existing membrane lipids and producing lipid mediators [11], [22]–[25]. The cleavage products such as lysophospholipids, non-esterified (free) fatty acids, diacyl- and monoacylglycerols or ceramides, together with the newly synthesized lipid molecular species, may be reinserted at different membrane sites inducing formation, segregation or rearrangement of membrane microdomains. Indeed, recently we reported that modulations in membrane fluidity and/or microheterogeneity achieved either by heat or a fluidizing agent resulted in pronounced and highly-specific alterations in the membrane lipid composition of B16 cells [25]. Additionally, we assume that, together with the retailoring of certain lipid molecular species and besides lipases or certain members of protein kinase C family [22], some preexisting subpopulations of Hsps may also become membrane-associated in cells preexposed to hyperfluidization stress. A subpopulation of Hsps is known to be present either on the surface or within the intracellular membranes [3], [6], [9], [26], [27] possibly associated with ordered microdomains (“rafts”) [7], [28], [29]. Moreover, Hsps have been shown to modulate major attributes of the membrane lipid phase state such as the fluidity, permeability, or non-bilayer propensity via their specific membrane lipid interactions [30], [31].

To gain further insights into the role of membranes during hyperfluidization-induced stress, we used K562 cells. These were heat-stressed and the resulting membrane organisation alterations were followed by Laurdan, the ideal fluorescent probe to study lateral structure of membranes in living cells by two-photon excitation fluorescence microscopy [32]. Laurdan's homogeneous distribution in membranes, and its lipid phase-dependent emission spectral shift offered a possibility of gaining novel information compared to that obtained using fluorescent probes which preferentially partition into specific membrane regions but whose fluorescence intensities and spectral maxima are generally insensitive to the lipid phase state [33]. Using the Laurdan-labelled K562 cells, spatially resolved membrane lateral packing and/or domain information could be obtained directly from the fluorescent images. These data have been compared with those obtained by “classical” approaches, which provide “bulk” information such as anisotropy or fluorescence lifetime measurements with 1,6-diphenyl-1,3,5-hexatriene (DPH)analogues and electron paramagnetic resonance (EPR) measurements with spin-labelled probes. Applying these methods in heat-primed vs. non-stressed cells or isolated plasma membranes provided important new information concerning the complex array of surface and intracellular membrane events initiated by HS.

## Results

### Laurdan two-photon microscopy reveals that heat stress causes spatially-distinct membrane re-organisation *in vivo*

To assess changes in the physical state of cell membranes after a temporary HS (42°C, 1 h), we analyzed Laurdan fluorescence using two-photon microscopy. Laurdan is an environmentally-sensitive fluorescence probe that exhibits an emission spectral shift depending on the lipid phase state, i.e., bluish in ordered, gel phases and greenish in disordered, liquid-crystalline phases. Laurdan distributes equally between lipid phases and does not associate preferentially with specific fatty acids or phospholipid headgroups. As a normalized ratio of the intensity at the two emission wavelengths regions, the generalized polarization (GP) provides a measure of membrane order, in the range between −1 (liquid-crystalline) and +1 (gel) [33], [34].

**Figure 1:**
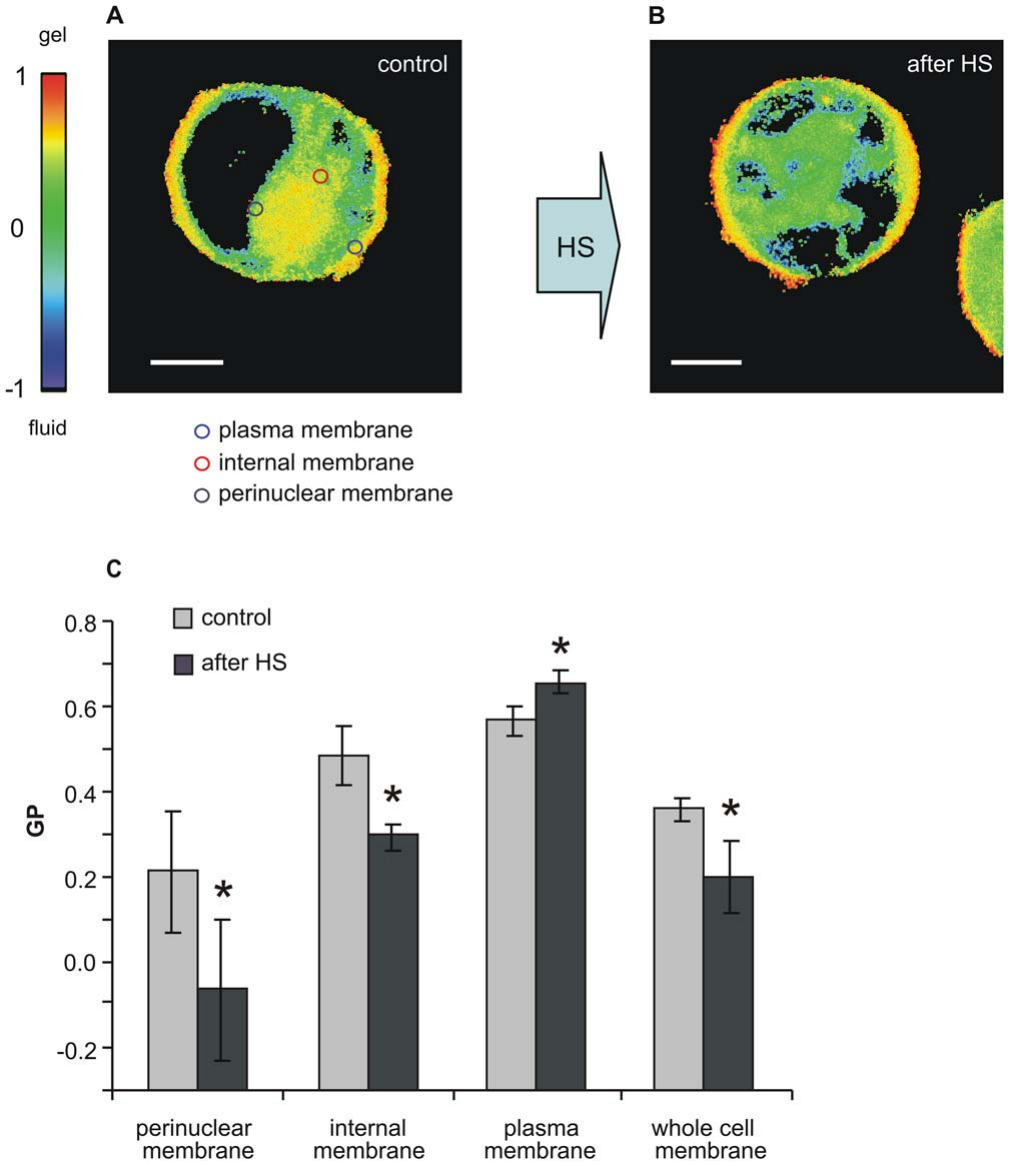
Heat-induced membrane changes visualised by Laurdan two-photon microscopy. K562 cells in RPMI medium were treated at 42°C for 1 h and imaged (**A**) before and (**B**) after HS at room temperature. The colour chart in Panel A represents Laurdan GP values as indicated. Bars in A and B, 6 µm. (**C**) Histogram of GP changes observed in whole cell and in subcellular (perinuclear, internal and plasma membrane) regions. An example set of ROIs used for quantification of subcellular GP distribution is given in panel A. Data are expressed as means ± SD, n = 30, *p<0.05 compared to the control, unpaired *t*-test.

**Figure 2:**
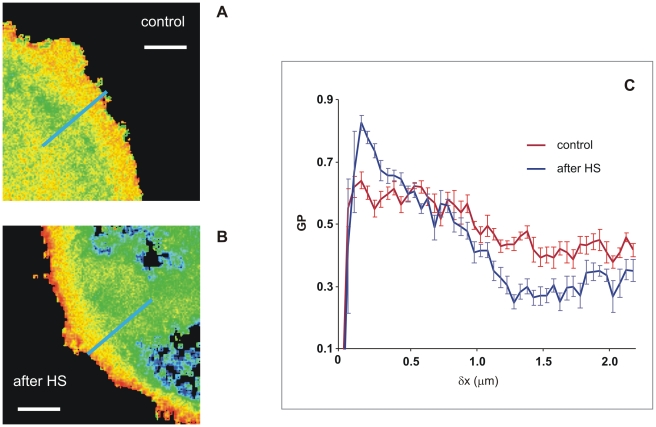
High resolution images of the general polarisation distribution at the cell border. K562 cells in RPMI medium were treated at 42°C for 1 h and imaged (**A**) before and (**B**) after HS at room temperature. Bars in A and B, 1 µm. (**C**) GP profile along blue lines shown in panels A and B. For other details see Figure 1.

In our experiments with Laurdan-labelled K562 cells, GP images revealed high-GP regions at the PM while low-GP regions were mainly found at internal and perinuclear membranes (Figure 1A) consistent with previous findings in various cell types [35]. When cells were heated at 42°C for 1 h and subsequently cooled down to 22°C for the measurement, on the whole the membranes became more disordered than in the non-stressed controls (Figure 1B), and this resulted in an average shift towards lower GP values (Figure 1C). A closer look also revealed that the PM and the endomembranes in K562 cells subjected to HS displayed contrasting behaviour. Indeed, our findings by Region-of-Interest (ROI) analysis demonstrated that the PM became more rigid, while the internal membranes, especially the perinuclear region became more fluid following heat treatment. The GP profiles of high resolution images clearly display the condensed cell surface and disordered inner membrane region in post-heat cells compared to control (Figure 2).

### Fluorescent polarisation shows “well-mannered” fluidity alterations in isolated membranes but anomalous responses *in vivo*

**Figure 3:**
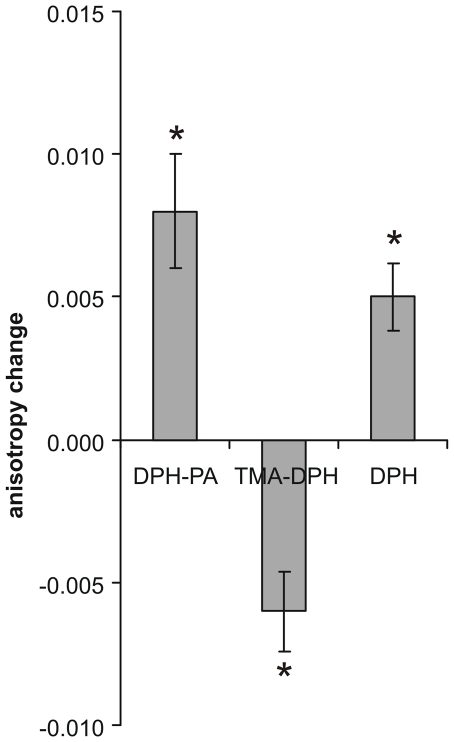
Anisotropy changes as detected by different fluorescent probes. K562 cells in RPMI medium were heat-treated at 42°C for 1 h or left at 37°C, harvested and labelled with DPH-PA, TMA-DPH or DPH. The fluorescence steady-state anisotropy measurement was performed at 37°C and 5 min of trace was averaged. The anisotropy differences were calculated relative to the 37°C control values. Data are represented as means ± SD, n = 4, *p<0.05, paired *t*-test.

In order to investigate the heat-induced membrane rearrangements further, DPH fluorescent probes with negatively or positively charged surface anchors, (1,6-diphenyl-1,3,5-hexatriene propionic acid (DPH-PA) or 1-(4-trimethylammoniumphenyl)-6-phenyl-1,3,5-hexatriene *p*-toluenesulfonate (TMA-DPH), respectively) and the non-anchored DPH itself were used. Cells were kept in growth medium during 1 h of HS at 42°C. They were then harvested and labelled at 37°C for 30 min (DPH) or 5 min (TMA-DPH, DPH-PA), times appropriate to the individual probes [15]. The changes in fluorescence anisotropy showed a fluidity decrease both with DPH-PA and DPH, but a fluidity increase with TMA-DPH, as depicted in Figure 3.

**Figure 4:**
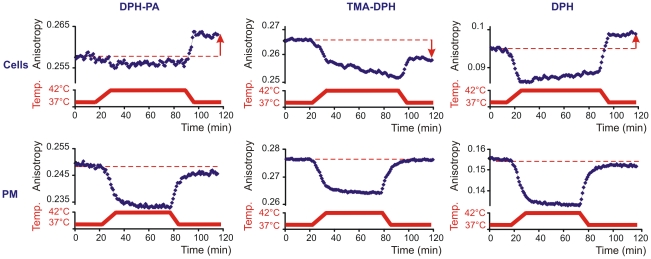
Fluorescence changes during heat treatment are different in cells compared to isolated plasma membranes. K562 cells or plasma membrane fractions (PM) isolated from untreated cells were labelled with DPH-PA, TMA-DPH or DPH and the fluorescence steady-state anisotropy (blue) was followed (representative traces are shown from n = 4 independent experiments). Cyclic temperature shift was applied (red). The arrows indicate the anisotropy difference at 37°C before and after 42°C HS.

To follow the time course of heat-induced membrane rearrangements, changes in membrane fluidity were measured in living cells and were compared with those obtained with PM isolates (as non-living, non-responding controls) in response to a heating/cooling cycle. As expected, with all three labels we saw a fluidity increase during the heating period with isolated PMs which returned completely to its initial value after restoring the incubation temperature to 37°C (Figure 4).

In contrast to this “normal” fluidity–temperature profile, membrane fluidity measurements conducted on living cells displayed an anomalous response to heat stress. The negatively charged probe DPH-PA revealed no change in the lipid order during heating. An anisotropy increase was detectable, however, as soon as the temperature was decreased back to 37°C and became constant at this temperature. Monitoring membrane rearrangements with TMA-DPH revealed a strong membrane fluidization upon temperature increase and a further slow, steady increase of membrane disordering during 1 hour exposure to 42°C. Upon returning back to normal (37°C) growth temperature an ordering effect was observed, although the anisotropy did not reach the initial value. The DPH probe measured a prompt fluidization following shift to 42°C after which a slight rigidization took place gradually at this temperature. When returned to 37°C, the DPH anisotropy value detected in cells was higher than that measured prior to HS (Figure 4).

### Benzyl alcohol-induced fluidization also shows distinct differences between isolated plasma membranes and cells *in vivo*

**Figure 5:**
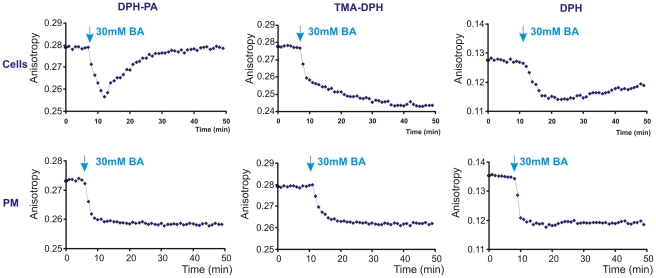
Changes in anisotropy upon benzyl alcohol addition also differ between cells and isolated plasma membranes. K562 cells or plasma membrane fractions (PM) isolated from untreated cells were labelled with DPH-PA, TMA-DPH or DPH and the fluorescence steady-state anisotropy was followed at 37°C (representative traces are shown from n = 4 independent experiments). BA was administered as indicated (blue arrows).

To gain further insights into the kinetics of hyperfluidization-induced membrane rearrangements, the effect of the chemical membrane fluidizer BA at a previously selected concentration (30 mM), which mimics various effects of 42°C heat treatment [15], was also tested. Transient fluidization was seen in living cells as a consequence of BA treatment, followed by an exponential compensatory decay reported by DPH-PA and, to much lesser extent, by DPH. However, using TMA-DPH as a probe we detected only fluidization. This reached an almost constant level after about 20 min. BA administration resulted in a very pronounced fluidization on isolated PMs with all three probes, as expected (Figure 5).

It is important to note that we repeated the in vivo experiments using DPH and its analogues using B16 mouse melanoma, L929 mouse fibroblastoid, WEHI164 mouse fibrosarcoma, HeLa human epithelial carcinoma and freshly isolated mouse spleen cells with strikingly similar changes in the fluidity profiles observed upon heat or BA treatment. This shows the broad applicability of our data with K562 cells to other cell types.

### Heat stress causes changes in membrane heterogeneity as detected by lifetime distribution

**Figure 6:**
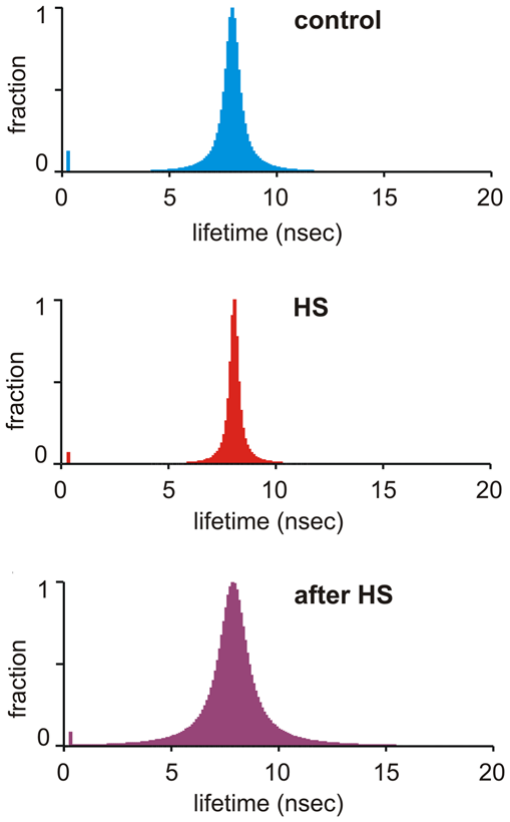
Heat-induced membrane heterogeneity changes followed by DPH lifetime distribution in K562 cells. Cells were labelled with DPH. Phase and modulation data were collected using 9 modulation frequencies ranging from 2 to 180 MHz. Measurements were performed at 37°C, during 1 h HS at 42°C and in the post-heat phase after returning to 37°C (representative results are shown from two independent experiments).

The DPH lifetime value has been shown to be sensitive to the membrane's physical state, this latter causing a change in the dielectric constant of the probe's environment itself. Indeed, the dielectric constant is the physical property to which DPH fluorescence lifetime responds [36]. In a continuous distribution of DPH lifetime values, the centre value yields information about the average polarity of the entire fluorophore environment, while the full width at half maximum (abbreviated to ‘width’, in the following text) gives a measure of the heterogeneity of this environment. The DPH lifetime distribution was measured in K562 cells before, during and after 1 h of HS at 42°C (Figure 6). While the centre values remained unchanged during the heating/cooling cycle, the width changed in the different conditions: when compared to the initial state, the width decreased with heating, and increased again, to values higher than initial ones, in the post-heating phase (cells at 37°C after HS). This indicates a transient relative decrease in membrane heterogeneity during HS, followed by a recovery to a significantly higher membrane heterogeneity in the post-heating phase.

### DPH analogues distribute differently within cells

**Figure 7:**
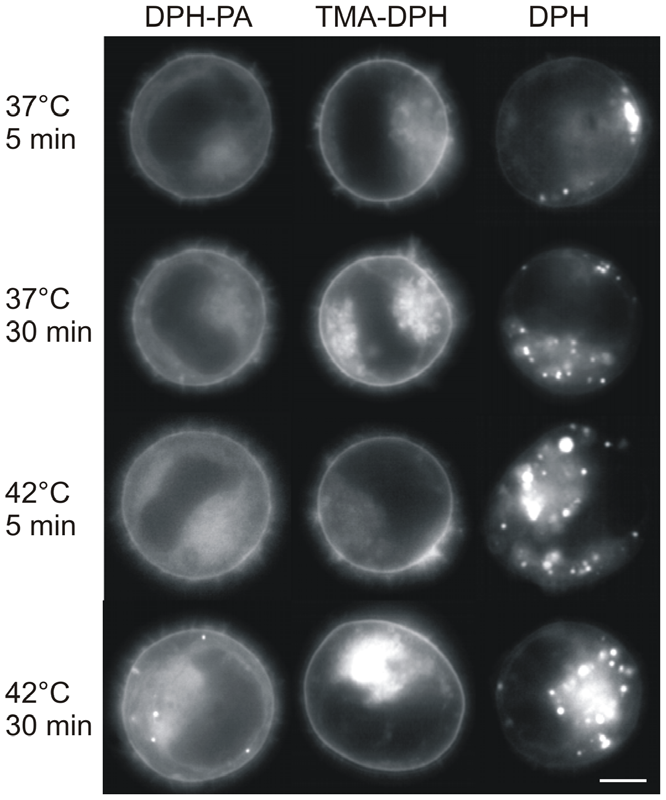
Different fluorescent probes show distinct patterns of localization in K562 cells. Cells were incubated with different DPH analogues at 37°C or 42°C for 5 or 30 min as indicated. Fluorescence video microscopic images were recorded within 2 min following the incubation (representative results are shown from n = 3 independent experiments). Bar, 6 µm.

**Figure 8:**
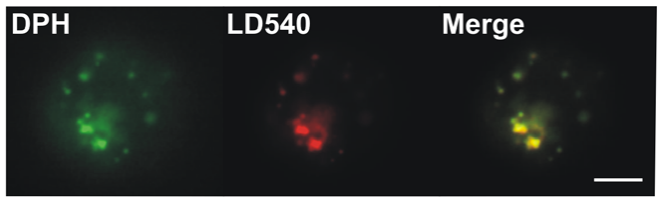
Colocalization of DPH with lipid droplets in K562 cells. Cells were double-stained with DPH and LD540 (which stains LDs) and examined with a CytoScout fluorescence microscope. Bar, 6 µm.

Data in the literature on the subcellular distribution of the probes is somewhat controversial (see details in Discussion). Therefore, in order to visualise which subcellular compartments were labelled by the probes, a fluorescence microscopic study was performed. Images were collected after 5 and 30 min labelling periods at different temperatures with DPH and its derivatives (Figure 7). We conclude from these data that individual DPH analogues label different cellular compartments to varying extents. DPH-PA showed a bright PM staining, but the nuclear membrane and an internal lamellar structure (probably ER) were labelled as well. The fluorescence signal for the PM was the most obvious with TMA-DPH which also showed fainter, diffuse and vesicular internal structures. Surprisingly, labelling was less intense with DPH for all the cellular membranes and the brightest signals originated from discrete spots, which we assumed to be lipid droplets (LD). To test the latter conclusion, a colabelling experiment was conducted. Cells were labelled with DPH and a lipid droplet specific dye LD540 [37]. Merging of the images revealed that the spots stained with DPH were almost completely colocalized with LDs stained with LD540 (Figure 8).

### EPR studies confirm that heat stress causes membrane structural re-arrangements

**Figure 9:**
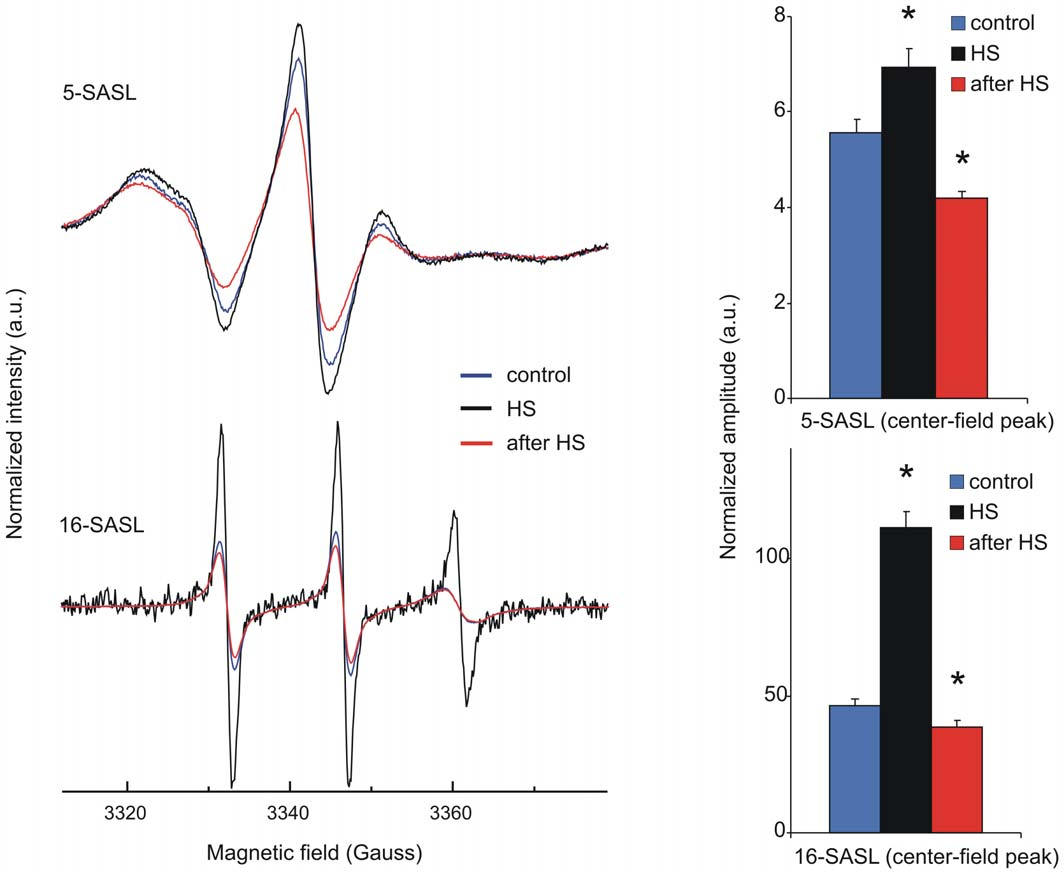
Spin-labelling of K562 cells reveals changes in membrane rigidization following heat stress. EPR spectra of K562 cells labelled with 5- (top) and 16-SASL (bottom) are shown. Spectra were recorded at 37°C (blue lines, control), during 1 h HS at 42°C (black lines, HS) and after returning to 37°C (red lines, after HS). The spectra are normalised so that they represent the same number of spins. Total scan range is 10 mT. The corresponding bar graphs show the normalized amplitudes of the center-field ^14^N hyperfine EPR lines (m_I_ = 0). Data are expressed as means ± SD, n = 3 (independent preparations), *p<0.05 compared to 37°C control, unpaired *t*-test.

The characteristic sensitivity of spin-label EPR spectra to membrane dynamics provides data on the membrane environment of nitroxide-labelled stearic acids [38], [39]. The 5- and 16-(4′,4′-dimethyloxazolidine-*N*-oxyl)stearic acid spin labels (5- and 16-SASL) partition into biomembranes from the aqueous phase with high affinity and locate their paramagnetic nitroxyl group close to the lipid headgroup and the centre of the bilayer, respectively (see e.g., [40]). EPR spectra of K562 cells labelled with 5- or 16-SASL were recorded before, during and after HS and were compared. While there were no remarkable changes in terms of hyperfine splitting, we observed significant alterations in the line widths at both vertical locations, probed by 5- and 16-SASL, respectively (Figure 9). Raising the temperature from 37°C (blue lines) to 42°C (black lines) increased the amplitude of the EPR lines in a depth-dependent manner due to motional line-narrowing, as expected (Figure 9). However, in the post-heat state when, after 1 h HS at 42°C the measurements were carried out at 37°C (red lines), we saw a line-broadening effect and a decrease in the line heights, indicating rigidization, compared to the pre-heat state for both 5- and 16-SASL spin labels. To have an estimate of this line broadening, the normalized amplitude values (maximum line height normalised to the total spin label intensity, [41]) were calculated and a similar relative change was found between the pre- and post-heat state for both labels (ratios of 0.76 and of 0.83 for 5- and 16-SASL (pre-heat:post-heat) center-field peaks, respectively, Figure 9, bar graphs).

## Discussion

### Different probes reveal different aspects of membrane organisation

Previously we demonstrated that non-proteotoxic membrane perturbants are able to lower the temperature threshold for the heat shock response, i.e. to enhance the expression of the Hsps in mammalian cells [15], [16]. To explore membrane changes as a consequence of hyperfluidization stress, membrane organisation alterations were followed by different techniques. All fluorescence and paramagnetic probes used in this study were able to detect membrane changes upon heat and/or BA stress. It is intriguing, however, as to why the membrane order imaged by Laurdan and the membrane fluidity detected by different DPH analogues or spin-labelled probes show such distinct features (Table 1). There are many reasons for environmental heterogeneity with respect to the properties of probes in individual environments, e.g. the distribution of probes among cellular membranes and LDs, preference for the inner or outer leaflet of the PM, localization in a defined position (depth), attraction to or repulsion from charged environments or partition into specific lipid-protein or lipid-lipid organizations (domains or clusters) [42]. Therefore, any conclusion from experiments in which only a single probe is used should therefore be drawn with great care and no general interpretations of membrane fluidity should be proposed.

**Table 1:** Characteristic properties of different probes used in the present study.

	Membrane localization	Charge	Effect seen at post-stress state^a^
Laurdan	whole cell membrane	no	overall fluidization
Laurdan	PM (confocal microscopy)	no	rigidization
Laurdan	endomembranes (confocal microscopy)	no	fluidization
TMA-DPH	PM and PM-like endocytic compartments	positive	fluidization
DPH-PA	PM and endomembranes	negative	rigidization
DPH	mainly LDs	no	rigidization
5-SASL	PMendomembranes?(not in mitochondria)	negative	rigidization
16-SASL	PMendomembranes?(not in mitochondria)	negative	rigidization

a compared to the initial pre-stress state (control at 37°C). PM, plasma membrane; LD, lipid droplet.

### Laurdan shows contrasting temperature-induced changes in fluidity in different cellular membranes

By using Laurdan two-photon microscopy we demonstrate clearly an increased order (or a decreased fluidity) in PMs in contrast to a decreased membrane order (or an increased fluidity) within the intracellular membranes, particularly perinuclear, of cells preexposed to HS. This result was made possible thanks to the spatial resolution of Laurdan GP microscopy images. Indeed, in the whole cell, when the GP values were averaged over all cell membrane compartments, we simply obtained a decreased order, an overall fluidization effect after HS which clearly did not reveal the subtle nature of the changes.

### DPH analogues distribute subcellularly depending on their chemical structure

The cellular and intramembrane localization of DPH analogues has been widely studied. They have been thought to report at least in part from the PM [19]. Indeed, TMA-DPH strongly incorporates into PM within 5 min (Figure 7). The internalization of this probe can mainly result from endocytosis, since the absence of TMA-DPH from non-endocytic compartments has been noted [43]. From TMA-DPH fluorescence anisotropy assays it was concluded that membrane fluidity remains identical in the PM and in the endocytic compartments [44]. Comparing our microscopic images with those obtained by the Kuhry-group and taking into account the constant nature of the anisotropy during the first 20 min trace at 37°C (Figure 4), corroborates the above-mentioned view. Our results obtained with TMA-DPH reported an increased fluidity in PM (and PM-like endocytic compartments) following HS. Post-heat fluidization of PM measured by TMA-DPH was found also in liver cells after 42°C heating by Revathi and coworkers [19] and in several other cell lines following a severe 45°C thermal challenge [21]. In contrast to TMA-DPH, the negatively charged DPH-PA reporting from PM and endomembranes (including the perinuclear region as well) (Figure 7) displayed a less fluid environment after HS (Figs. 3, 4). Importantly, the anisotropy values in the post-stress relaxation period at 37°C (Figure 4) displayed the same characteristics as were seen with post-heat labelling (Figure 3), i.e. anisotropy increase with DPH-PA and decrease by TMA-DPH. It seems, therefore, that the short (5 min) or long (ca. 60 min) labelling periods did not influence the outcome of anisotropy values with these probes.

Besides their different localization, positively or negatively charged probes are thought to display leaflet specificity. However, several partly contradictive conclusions appear in the literature. Kitagawa and coworkers [45] stated that the quaternary ammonium cation TMA-DPH binds first to the outer layer of the PM and then gradually penetrates the cytoplasmic side, distributing in both membrane leaflets but mainly to the inner leaflet, whereas the fluorescence anisotropy of anionic DPH-PA, according to these authors, reflects the fluidity of outer leaflet of the PM because of electric repulsion from the region, where acidic phospholipids are present. In contrast, the Schroeder group drew the opposite conclusion to explain their findings by the “charge similarity” principle: TMA-DPH appears to selectively localize in the outer leaflet, while the negatively charged DPH-PA appears to localize in the PM inner leaflet [46]. Nevertheless, the Kurhy group [43] reported that TMA-DPH, once incorporated into the peripheral membranes, is retained in the external leaflet of the bilayer. It is certainly conceivable, whatever the leaflet specificity of these probes is, that they prefer different lipid microenvironments due to their electric charge. Accordingly, the free fatty acid derivative (i.e. negatively charged) EPR probes may partition similarly to DPH-PA to the membranes reporting most probably from the same charge-specific microenvironments. Although the localization of EPR probes cannot be studied directly, the observed line broadening effects were not dependent on the depth of the reporter moiety as we found a similar relative change in the normalised amplitude as a result of HS by both spin labels (Figure 9). The line broadening was too small to determine which possible factors (e.g. changes in rotational dynamics or spin-spin interaction) contributed significantly, but some structural rearrangements, such as lateral reorganisation of membrane domains or an overall decrease in membrane fluidity [47] could explain the observed spectral effects. Taken together, the negatively charged fluorescent and EPR probes regardless of their vertical localization in the bilayer, reported a less fluid environment in the post-heat state.

### DPH itself partitions into lipid droplets

In contrast to its analogues, DPH is not strictly localized in the cellular membranes but partitions into LDs with high efficacy as well (Figures 7 and 8). This fact has been largely neglected in the literature despite some research data showing the masking effect of triacylglycerols on whole cell membrane anisotropy measurements with DPH [48]–[51]. Until recently, LDs, which occur in almost all mammalian cells, were considered inert storage sites of energy dense fats. Nowadays, however, LDs are increasingly considered dynamic functional organelles involved in many intracellular processes like lipid metabolism, vesicle trafficking, and cell signalling [52]. DPH reported a fluidity decrease in the post-heat state of K562 cells (Figure 4) in agreement with the results of Revathi et al. [19] on liver cells, but we suggest that this is not attributable to the PM changes, but rather to the rearrangement of intracellular membranes and/or LDs. The DPH fluorescence lifetime measurement showed no change in the center value, thus the average polarity of the entire fluorophore environment remained unaltered by HS. On the other hand, a much broadened lifetime distribution in samples derived from the post-heating phase reflected more heterogeneous microenvironment for the probe. These results suggest that LDs also undergo heat-induced remodelling.

### Cells modify the fluidization seen in isolated membranes

Beyond the above heat-induced membrane rearrangements detected in the post-heat state by DPH and its analogues, the time course of the reorganization revealed anomalous (and probe-dependent) responses of K562 cells to hyperfluidity stress. Behind the unchanged fluidity observed by DPH-PA upon HS, treatment with the chemical fluidizer BA revealed two-phase kinetics composed of a rapid fluidization followed by an exponential fluidity compensation. This suggests that BA enters the membranes rapidly, while heating up the cuvette takes a longer time, long enough to maintain the anisotropy constant by continuous compensation during heating. Rearrangements revealed by TMA-DPH displayed an instantaneous membrane disordering upon both heat and BA stresses followed by a further reduction of anisotropy. A similar time-dependent gradual decrease in the TMA-DPH anisotropy was seen during heat or alcohol treatment of Jurcat cells [11]. The anomalous behaviour of living cells is in sharp contrast to the “well-mannered” fluidity–temperature profile of isolated PM, thereby strongly indicating an active process, e.g. upregulated lipid enzyme function and/or protein translocation or transport targeting the membranes in intact cells. Furthermore, the strikingly similar changes in the fluidity profiles upon hyperfluidity treatment observed in several other cell lines *in vivo* suggest that the membrane rearrangement induced by the “instant membrane perturbation” is a general phenomenon. It may, therefore, allow us to discuss and interpret associated biochemical and biophysical phenomena in a more general context.

### The probes detect changes in ordered membrane domains following stress

Laurdan microscopy has been proven in various studies to be capable of visualizing lipid structure and raft domains in living cells [53] and showing the coalescence of ordered domains in specific membrane morphologies, such as filopodia in macrophages [53], lamellipodia in neutrophils [54] or immunological synapses in T lymphocytes [55]. Analogously, the higher GP values reported from PM of K562 cells in this study can be attributed to coalescence and/or de novo formation of ordered, raft-like domains as a consequence of stress. These results are in full accordance with our previous data obtained in B16 cells by using the fluorescein ester of polyethylene glycol-derivatized cholesterol (fPEG-Chol), which specifically recognizes sterol-rich membrane domains and colocalizes with various raft markers. When B16 cells were exposed to heat or BA treatment, the cholesterol-rich surface membrane microdomains fused into larger platforms upon both treatments and this change persisted for hours after ceasing the stress [16]. Furthermore, Ca^2+^ loading of erythrocytes was coupled to an elevated lipid order [56]. We also demonstrated previously in K562 cells [15], that the intracellular Ca^2+^ level is ubiquitously upregulated in heat-stressed cells [57]. This intracellular Ca^2+^-rise may play a role in the ordering of PM as detected by Laurdan in response to HS (Figure 1).

It is known that elevated ceramide levels can rapidly displace cholesterol from membrane/lipid-“chol-rafts” to form “cer-rafts” [58]–[60]. Enhanced ceramide generation was documented by Moulin and coworkers [11] in a variety of cell types (HL60, U937, Jurkat and Jurkat A3 cells), and a key feature of the lipid remodelling due to heat or BA-induced membrane perturbation was also the accumulation of ceramide in B16 cells [25]. Furthermore, Al-Makdissy and coworkers [61] measured DPH and TMA-DPH anisotropy during the sphingomyelinase digestion of PM. They found that DPH and TMA-DPH anisotropy dropped when ceramide was produced. In other words, the probes found themselves in a more disordered lipid environment upon ceramide generation. Megha and London [62] showed that this drop in anisotropy is due to displacement of DPH, and probably TMA-DPH, from the ordered ceramide-rich rafts to the disordered lipid regions of the bilayer. Gallegos et al. compared the fluidity of PM and detergent-free raft fractions isolated from murine L-cells using TMA-DPH and DPH-PA probes [46]. Interestingly, TMA-DPH reported the isolated raft to be more fluid than the PM, while DPH-PA reported the opposite. It was also unambiguously exemplified in the present study that the microenvironmental changes might be sensed substantially differently or even oppositely by structurally different probes, i.e. fluidization of PM was measured by TMA-DPH as opposed to the increased packing observed by Laurdan. The two phenomena can be intimately linked most probably to the extrusion of TMA-DPH from the newly formed or restructured ordered PM domains with stress, resulting in an overall increase in TMA-DPH fluidity. DPH-PA and the EPR probes, despite the differences in the depth of the probe location, reported an overall rigidization of the cellular membranes after HS, while Laurdan displayed an average fluidization. An explanation could be that the negatively charged probes are more sensitive to the rigidization originated from the stress-induced ordered domain formation. This view is supported by the fact that the acidic derivative of Laurdan, C-laurdan has been suggested to be better sensor of lipid rafts compared to its parent molecule [63], [64].

### Membrane heterogeneity can influence thermo-sensitivity or tolerance

The above-described membrane changes can also be interpreted in relation to thermosensitivity or thermotolerance. In a comparative study heat-resistant and -sensitive mutants of CHO cells, mouse fibrosarcoma variants and Crandall feline kidney cells were challenged at 45°C [21]. It appeared that the level of post-heat fluidization detected by TMA-DPH can be a marker for the heat sensitivity of the cells.

Rapid remodelling of cell membranes following mild membrane fluidization can directly contribute to the acquisition of cellular stress tolerance [12] or indirectly is involved in the upregulation of Hsp synthesis [16], [25]. The enhanced formation of rigid domains in surface membranes and the parallel elevation of molecular disorder within the internal membranes, firstly demonstrated here in K562 cells preexposed to mild HS, is also known to be accompanied by a massive formation of Hsps without any detectable loss of cell survival [15]. Recently, the localization of Hsp72 and Hsp60 was also seen to be affected by certain membrane fluidizing and HS treatments causing a decrease in intracellular Hsps with a simultaneous increase in surface Hsps [7]. Moreover, it was demonstrated that membrane fluidizing treatments, like hyperthermia can either enhance or inhibit the cytotoxicity of specific apoptosis inducers depending on cell type, cytotoxic drug or the stage of drug treatment at which the fluidizing treatment is applied [7]. Consequently, decision-making between cell protection and cell death may involve similar processes; however, the contribution of individual elements can be different thereby leading to the reorganisation of plasma and intracellular membranes to various extents. Microscopic imaging of fluidization-induced membrane remodelling together with parallel monitoring of signal transduction events originating from membranes, Hsp synthesis and protein translocation has broad implications for understanding the mechanism of cell killing and survival. This, in turn could have an important application in developing better strategies for cancer therapy.

## Materials and Methods

### Materials

RPMI-1640 medium and 5- and 16-(4′,4′-dimethyloxazolidine-*N*-oxyl) stearic acid spin labels (5- and 16-SASL) were purchased from Sigma (Steinheim, Germany). 1,6-diphenyl-1,3,5-hexatriene (DPH), 1,6-diphenyl-1,3,5-hexatriene propionic acid (DPH-PA), 1-(4-trimethylammoniumphenyl)-6-phenyl-1,3,5-hexatriene p-toluenesulfonate (TMA-DPH) and 6-dodecanoyl-2-dimethylaminonaphthalene (Laurdan) were from Molecular Probes, Inc. (Eugene, OR). The LD-specific dye LD540 was a generous gift from Professor Christoph Thiele (Bonn, Germany). Benzyl alcohol (BA) of analytical grade was from Merck (Darmstadt, Germany). All other chemicals were purchased from Sigma and were of the best available grade.

### Cell culture

K562 cells (ATCC: CCL-243) were cultured in RPMI-1640 medium, supplemented with 10% fetal calf serum and 2 mM glutamine in a humidified 5% CO_2_, 95% air atmosphere at 37°C and cultured for a few passages.

### Fluorescence anisotropy

The plasma membrane fraction of K562 cells was isolated according to Maeda et al. [65]. Isolated plasma membranes were labelled in 10 mM Tris, 10 mM NaCl (pH 7.5) with 0.2 µM DPH or its derivatives, TMA-DPH and DPH-PA at a molar ratio of about 1∶200 probe/phospholipid for 5 minutes. For in vivo fluidity measurements, K562 cells were labelled with 0.2 µm DPH for 30 min, or with DPH-PA and TMA-DPH for 5 min. Steady-state fluorescence anisotropy was determined in a PTI spectrofluorometer (Quanta Master QM-1, Photon Technology International, Inc., Princeton, NJ, USA). Excitation and emission wavelengths were 360 and 430 nm, respectively (5-nm slits) [31].

When the temperature dependence of fluidity was followed, the temperature was maintained at 37°C for 5 min, programmed at 0.4°C/min from 37°C to 42°C, maintained isothermally for 1 h, cooled down gradually (0.4°C/min) to 37°C and held for 5 min.

The time course of the fluorescence anisotropy upon addition of BA was performed as follows: cells were incubated at 37°C for 5 minutes and BA (30 mM) was introduced into the cuvette. The anisotropy data were collected every 30 s.

### DPH lifetime distribution studies

K562 cells were labelled with 0.2 µM DPH for 30 min. Time-resolved emission was measured using the K2 phase fluorometer (ISS Inc., Champaign, IL) [66]. The excitation source was a He-Cd laser (λ = 325 nm). Phase and modulation data were collected using 9 modulation frequencies ranging from 2 to 180 MHz. Lifetime measurements were performed using a reference solution of 1,4-bis(5-phenyloxazol-2-yl)benzene in ethanol (τ = 1.35 ns). Emission was observed through a KV370 cutoff filter (Schott Glass Technologies Inc., Duryea, PA). Data were analyzed using the Globals Unlimited software (Laboratory for Fluorescence Dynamics, University of California at Irvine, CA).

### EPR studies

Cells were kept at 37°C (control) or heat-shocked at 42°C for 1 h and pelleted by centrifugation at 400 *g* for 5 min and subsequently washed twice in excess volume of phosphate buffered saline pH = 7.4 (PBS) at 37°C. The final cell pellets were resuspended in PBS at a concentration of 1.2×10^7^ cells/ml. For EPR measurements an aliquot of 400 µl cell suspension was used and cells were spin labelled. Briefly, 2.5 µl of 5-SASL (2.5 mg/ml in ethanol) or 5 µl of 16-SASL (1 mg/ml in ethanol) spin label was added to the cell suspension, kept for 5 min at room temperature and occasionally vortexed. Cells were pelleted by centrifugation at 400 *g* for 5 min and the pellet introduced into 1 mm diameter EPR glass capillary. Capillaries were further centrifuged in a bench top centrifuge (400 *g*, 5 min) and the top layer was removed to obtain a sample length of 5 mm in each capillary. EPR spectra were measured in a Bruker (Rheinstetten, Germany) ECS 106 X-band spectrometer equipped with a TE102 cavity and a nitrogen gas flow temperature regulator at 37°C or 42°C. Microwave power was 2 mW and 100 kHz field modulation with an amplitude of 0.1 mT was used for first-harmonic detection. Spectra were centred around 335 mT and scanned over a field width of 10 mT. For each sample, two spectra were accumulated with 20.5 ms averaging at each of the 1024 field steps. The time elapsed from labelling the sample and recording the spectra was marked and comparisons were made between spectra recorded at the same time intervals, to avoid any mismatch between the redistribution of the spin probe with time in the live cells. The spectra were recorded within the time interval where the rate of reduction of the doxylstearate due to internalization followed by mitochondrial reduction of the probe was negligible [67]. Peak heights and positions were measured using the corresponding routines in the Bruker software as well as the numerical integration of the spectra after baseline correction.

### Laurdan two-photon microscopy

Laurdan labelling was performed directly in the cell culture media. 10 µl of Laurdan stock solution was added per ml of RPMI medium. The stock Laurdan solution concentration was 1 mM in dimethyl sulfoxide, and it was renewed every three weeks. After 30 min of incubation in the dark at room temperature, the cover glass was washed once with RPMI and mounted upon a microscope slide.

Laurdan intensity images were obtained with an inverted confocal microscope (DMIRE2, Leica Microsystems, Germany) using a 63× oil immersion objective (NA 1.4) under excitation at 800 nm with a mode-locked Titanium-Sapphire laser (Chamaleon, Coherent, Santa Clara, CA). Internal photon multiplier tubes collected images in an eight bit, unsigned images at a 400 Hz scan speed. Laurdan intensity images were recorded simultaneously with emission in the range of 400–460 nm and 470–530 nm and imaging was performed at room temperature.

For image analysis the generalized polarization GP, defined as
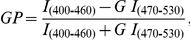
was calculated for each pixel using the two Laurdan intensity images (I_(400–460)_ and I_(470–530)_) [35]. The calibration factor G was obtained from the GP values of solutions of Laurdan in DMSO. The G factor had ∼2% variation across the imaging area. GP images (as eight-bit unsigned images) were pseudocoloured in ImageJ. Background values (defined as intensities below 7% of the maximum intensity) were set to zero and coloured black. GP histogram values were determined within multiple circular Regions-of-Interest (ROI) with 20 pixel diameter (Area 316 pixel) for different membrane regions. ROI for PM have been chosen along the contour profile of the cell, the perinuclear ROI along the contour profiles of the nuclei, the ROI for internal membranes (except the perinuclear region) in the rest of the cell. n = 10 ROI were measured for each membrane region within one cell. 3 control and 3 heat-stressed cells were analysed.

Line profiles and analysis of acquired images were performed with ImageJ (http://rsbweb.nih.gov/ij/).

### Fluorescence microscopy

K562 cells were suspended in PBS and labelled with 2 µM DPH-PA, TMA-DPH or DPH for 5 min or 30 min at 37°C or 42°C as indicated. In the case of double-labelling, cells were labelled with a mixture containing 2 µM DPH and 30 nM LD540 for 30 min. Images were taken with a CytoScout fluorescent microscope (Upper Austrian Research GmbH, Linz, Austria) using a 100× objective, D365/10 and D405/30 filter for exitation and emission of DPH and HQ532/70 and HQ 600/40 filters for excitation and emission of the LD540 probe.

### Statistics

Data are presented as mean ± SD and compared using Student's *t*-test (paired or unpaired; details are specified in the Figures).
